# Draft genome sequences of *Megasphaera paucivorans*, a species of strict anaerobic beer spoiling cocci

**DOI:** 10.1128/mra.00404-25

**Published:** 2025-09-08

**Authors:** Manuel J. Arnold, Matthias A. Ehrmann, Yohanes N. Kurniawan, Koji Suzuki, Wolfgang Liebl

**Affiliations:** 1 Chair of Microbiology, Technical University of Munich, Freising, Germany; 2 Asahi Quality and Innovations Ltd, Moriya, Ibaraki, Japan; Wellesley College, Wellesley, Massachusetts, USA

**Keywords:** beer spoilage, *Megasphaera paucivorans*, genome, obligate anaerobe

## Abstract

The only two commercially available strains of *Megasphaera paucivorans* were cultivated and their genomes sequenced. As recurring beer spoiling bacteria, they cause unwanted turbidity and unpleasant odors. Their genomes harbor a number of putative defense mechanisms explaining their much-needed resilience to survive in the brewing environment.

## ANNOUNCEMENT

Beer-spoiling *Megasphaera* sp. is an economic and recurring issue for the brewing industry (1, 2). Spoilage by these bacteria is coined by off-flavors, such as acetic acid, caproic acid, iso- and n-butyric acid, propionic acid, iso- and n-valeric acid, acetoin, and H_2_S (3, 4). As of today, there are only two publicly available strains (DSM 16981^T^ and BEL B) since its discovery (4). Despite its initial isolation source, which is spoiled beer from Italy with an alcohol content of 5% (v/v) and a pH of 4.3 (4), both strains showed growth in up to 6.0% (v/v) ethanol and a pH as low as 4.0.

The strains were obtained from the German strain collection, DSMZ (German collection of microorganisms and cell cultures) and the Belgian strain collection, BCCM/LMG (Belgian Coordinated Collections of Microorganisms/LMG Bacteria Collection). Both were cultivated at 30°C under anaerobic conditions for 5 days using modified PYG medium (DSM 104). DNA was isolated using the E.Z.N.A. DNA/RNA Isolation Kit (Omega Bio-tek, Inc., Norcross, GA, USA) according to the instruction manual with the modification of incubating the sample in the elution buffer heated to 65°C for 5 min prior to elution.

Sequencing was done using Illumina NovaSeq 6000, and the library was prepared using Illumina DNA Prep Kit (Illumina, San Diego, CA, USA) to generate short reads (S4 PE150 XP; paired-end 2 × 150 bp; Eurofins Genomics Europe Sequencing GmbH, Konstanz, Germany) with 5,042,830 total reads and 1,512,849,000 total bases for DSM 16981^T^, as well as 4,896,040 total reads and 1,468,812,000 total bases for BEL B. The generation of long reads was achieved via single-molecule, real-time (SMRT) sequencing technology (PacBio Sequel II, Functional Genomics Center Zurich, ETH Zurich, Switzerland). The library was created with an insert size of 8 to 12 kb, delivering at least 200 Mb of raw data from two SMRT cells (1 × 120 min movies) using the P4-C2 chemistry. SMRT analysis (v 2.2.0.p2) and hierarchical genome assembly process (HGAP) were applied for assembly (5). The N_50_ of the genomes were 2,931,609 for DSM 16981^T^ and 2,997,787 for BEL B. The hybrid assembly was executed via Unicycler (v. 0.5.0) (6) using short- and long-read data, and the genome sequences were annotated using the National Center for Biotechnology Information non-WGS annotation pipeline (7–9).

The total sequence length and the main genome statistics are listed in Table 1.

**TABLE 1:** General and genome stats of both sequenced *M. paucivorans* genomes

	DSM 16981^T^	BEL B
Source	Spoiled beer	Beer
Country	Italy	Italy
Date of sampling	2002	2010
Obtained from collection	DSMZ	BCCM/LMG
Total sequence length	2,931,609	2,997,787
Contigs	3	2
GC content	40.5%	40.5%
Genome coverage	505.0	487.9
Genes	2,802	2,822
Coding ratio	96.5%	96.5%
Genes coding for protein	2,704	2,723
5S rRNA operons	4	4
16S rRNA operons	4	4
23S rRNA operons	4	4
tRNAs	53	53
CRISPR arrays	2	2

Both genomes harbor genes for amino acid decarboxylases (arginine, ornithine) known to represent a functional acid stress tolerance mechanism. The genomes also contain a number of chaperones, proteases, and efflux transporters that could be involved in stress tolerance of many sorts. Several alcohol dehydrogenases can be found that could participate in the degradation of sugar alcohols present in beer or presumably even aid in stress response to ethanol. A key trait of the genus is the ability to degrade lactate, which is indicated by genes that are annotated as L-lactate dehydrogenase (NXG59_04225, NXG59_03710, NXG27_03725, and NXG27_04255) and L-lactate permease (NXG59_11530, NXG59_10695, NXG27_11785, and NXG27_10955). There are genes that show a resemblance to known hop transporters HorA (NXG59_08080 and NXG27_08335) and HitA (NXG59_01755, NXG59_12090, NXG27_01750, and NXG27_12345) that are known to confer hop tolerance (10, 11). Four of those genes that show resemblance to HitA are annotated to be a Nramp family transporter (NXG59_01755, NXG59_12090, NXG27_01750, and NXG27_12345) that could constitute another helpful barrier for hop stress, as it is described to facilitate iron and manganese uptake, which are scavenged by hop acids (12).

## Data Availability

The assembled genomes are available under accession numbers JANSUW000000000 (DSM 16981^T^ = TMW 2.2464) and JANSUA000000000 (BEL B = TMW 2.2489) with BioProject accession number PRJNA868947. The raw reads for DSM 16981^T^ (TMW 2.2464) are available under SRA accession numbers SRX28880805 and SRX29435980 and for BEL B (TMW 2.2489) under SRX28880807 and SRX29435981.
